# High‐elevation hypoxia impacts perinatal physiology and performance in a potential montane colonizer

**DOI:** 10.1111/1749-4877.12468

**Published:** 2020-07-30

**Authors:** Jérémie SOUCHET, Eric J. GANGLOFF, Gaëlle MICHELI, Coralie BOSSU, Audrey TROCHET, Romain BERTRAND, Jean CLOBERT, Olivier CALVEZ, Albert MARTINEZ‐SILVESTRE, Elodie DARNET, Hugo LE CHEVALIER, Olivier GUILLAUME, Marc MOSSOLL‐TORRES, Laurent BARTHE, Gilles POTTIER, Hervé PHILIPPE, Fabien AUBRET

**Affiliations:** ^1^ Station d'Ecologie Théorique et Expérimentale du Centre National de la Recherche Scientifique Moulis France; ^2^ Department of Zoology Ohio Wesleyan University Delaware Ohio USA; ^3^ Catalonia Reptile and Amphibian Rescue Center (CRARC) Masquefa Barcelona Spain; ^4^ Bomosa, Pl. Parc de la Mola Les Escaldes Andorra; ^5^ Pirenalia Encamp Andorra; ^6^ Nature En Occitanie Toulouse France; ^7^ Département de Biochimie Centre Robert‐Cedergren Université de Montréal Montréal Canada

**Keywords:** developmental plasticity, embryonic metabolism, high‐elevation hypoxia, locomotor performance, *Natrix maura*

## Abstract

Climate change is generating range shifts in many organisms, notably along the elevational gradient in mountainous environments. However, moving up in elevation exposes organisms to lower oxygen availability, which may reduce the successful reproduction and development of oviparous organisms. To test this possibility in an upward‐colonizing species, we artificially incubated developing embryos of the viperine snake (*Natrix maura*) using a split‐clutch design, in conditions of extreme high elevation (hypoxia at 2877 m above sea level; 72% sea‐level equivalent O_2_ availability) or low elevation (control group; i.e. normoxia at 436 m above sea level). Hatching success did not differ between the two treatments. Embryos developing at extreme high elevation had higher heart rates and hatched earlier, resulting in hatchlings that were smaller in body size and slower swimmers compared to their siblings incubated at lower elevation. Furthermore, post‐hatching reciprocal transplant of juveniles showed that snakes which developed at extreme high elevation, when transferred back to low elevation, did not recover full performance compared to their siblings from the low elevation incubation treatment. These results suggest that incubation at extreme high elevation, including the effects of hypoxia, will not prevent oviparous ectotherms from producing viable young, but may pose significant physiological challenges on developing offspring *in ovo*. These early‐life performance limitations imposed by extreme high elevation could have negative consequences on adult phenotypes, including on fitness‐related traits.

## Graphical abstract

Hypoxia affects embryo development by increased heart rate, stabilized egg mass and affects negatively phenotypes at short term. Juveniles transplanted from hypoxia to normoxia can't achieve the same swimming performance as juveniles from normoxia.

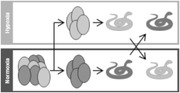

## INTRODUCTION

Climate envelopes are typically much narrower across altitudinal than latitudinal gradients (Loarie *et al*. 2009; Chen *et al*. 2011), fostering rapid migration along the elevational gradient as the climate warms (e.g. Walther *et al*. 2002; Parmesan & Yohe 2003; Bässler *et al*. 2013; Pauchard *et al*. 2016; Freeman *et al*. 2018). While lower‐elevation valleys may have provided refuge to many organisms during past glaciation events (Hewitt 1999; Tzedakis 2004), elevated areas may play a similar role for escaping global warming (Sinervo *et al*. 2018). As high‐elevation environments may represent climate refugia, it is important to identify constraints on upslope colonization. While it is well established that warming may promote range expansion toward higher altitudes, organismal function may be affected by the decrease in the oxygen partial pressure (for instance, oxygen availability is 25% lower at 2500 m above sea level [ASL] compared to sea level; Powell & Hopkins 2010; Storz *et al*. 2010). Yet, there are many unanswered questions regarding the effects of high elevation hypoxia on the ability of ectothermic vertebrates to colonize and adapt to these elevations.

The acute and chronic effects of high‐altitude hypoxia on organismal function seem to vary widely among taxa. Well documented in birds and mammals (Monge & Leon‐Velarde 1991; Beall *et al*. 2002; Storz *et al*. 2004; Lague *et al*. 2016), the acute effects of hypoxia commonly include hyperventilation, tachycardia, altitude sickness, and the down‐regulation of non‐essential physiological functions (such as digestion). These studies also demonstrate that chronic effects range from an alteration of cardiorespiratory pathways (increased lung and heart size, increased blood pressure), blood composition (increased hematocrit, increased hemoglobin concentration), and muscle performance (increased vascularization, increased amount of myoglobin and mitochondria) to effects on embryonic development, birth size, and early growth rates (Monge & Leon‐Velarde 1991; Beall *et al*. 2002; Storz *et al*. 2004; Lague *et al*. 2016). The consequences of high‐altitude hypoxia, induced by elevation, are less well known in reptiles. However, we can surmise that they might be similar to the consequences of high‐altitude hypoxia in birds and mammals or to underwater or underground hypoxia in reptiles. For instance, even short exposures to hypoxia can have lasting effects on subsequent growth and development of turtle embryos, including reduced mass at hatching, decreased oxygen consumption, and depressed metabolism, despite a comparable incubation period (Kam 1993; Cordero *et al*. 2017a). Incubation in hypoxic conditions is known to reduce embryo heart rates (in lizards: Cordero *et al*. 2017b; Kouyoumdjian *et al*. 2019), produce smaller juveniles with decreased growth rate during the first months of life (in alligators: Owerkowicz *et al*. 2009; in turtles: Wearing *et al*. 2015), and increase heart and lung size at birth (in alligators: Owerkowicz *et al*. 2009, and lizards: Cordero *et al*. 2017b). Chronic hypoxia specifically elicits changes in the cardio‐respiratory pathways (increases lung and heart size, higher blood pressure; Crossley & Altimiras 2005; Iungman & Piña 2013; Wearing *et al*. 2015; Cordero *et al*. 2017b), increases hematocrit and hemoglobin concentration (Vinegar & Hillyard 1972; Weathers & White 1972; Newlin & Ballinger 1976; González‐Morales *et al*. 2015; Lu *et al*. 2015; Gangloff *et al*. 2019), and alters muscle physiology (increases vascularization and myoglobin concentration; Jochmans‐Lemoine & Joseph 2018). Although many of the physiological and anatomical changes that accumulate under chronic hypoxia improve function under low O_2_ partial pressure (PO_2_; i.e. individuals show acclimation), these changes may only partially compensate for reduced oxygen availability. For example, low weights at birth and reduced growth in juveniles have been reported in a variety of vertebrate taxa, from humans (Monge & Leon‐Velarde 1991) to turtles (Wearing *et al*. 2015). Rats and mice showed delayed brain growth due to long‐term exposure to hypoxia (Golan & Huleihel 2006), cognitive effects which may be true in reptiles as well (Sun *et al*. 2014).

Predicting if and how animals will adapt to high altitude under global warming requires a detailed study of physiological, morphological, and behavioral responses to hypoxia across an altitudinal gradient in a species that undergoes upward range expansion. This knowledge is incomplete, particularly in snakes. The successful colonization of higher elevations in animals escaping warming temperatures depends on their ability to cope with lower partial pressure in oxygen so that they can: (i) move, acquire food, mate, and escape predators; and (ii) produce eggs (embryos) able to develop, hatch, and survive. Here, we focused on the latter as effective colonization (i.e. all former steps) depends on successful recruitment of offspring (Warner *et al*. 2012; Aubret 2013; While *et al*. 2015). To identify physiological, morphological, and behavioral alterations associated with altitude‐induced hypoxia, we performed an elevation transplant experiment utilizing a generally low‐elevation ectothermic species. In our experiment, we exposed eggs of the viperine snake, (*Natrix maura* Linnaeus, 1758; Colubridae), to two alternative incubation treatments: extreme high elevation (EHE, above current range limits, i.e. hypoxia) and low elevation (LE, native elevation, i.e. normoxia).

The viperine snake is a circum‐Mediterranean species that has been colonizing mountainous and lowland environments alternately in conjunction with historical warming and cooling cycles (Gómez & Lunt 2007). The Iberian Peninsula provided a glacial refuge during the Pleistocene and allowed the viperine snake to re‐colonize the Pyrenees and Western Europe from 12,000 years ago onward during the Holocene (Guicking *et al*. 2008). This aquatic species (Vacher & Geniez 2010) has been recorded up to 1000 m ASL in France (Aubret *et al*. 2015) and 1500 m ASL in Spain (Martinez‐Rica & Reiné‐Viñales 1988; Santos 2015). We collected gravid females from low elevation (475 m ± 43 m ASL), and using a split‐clutch design, incubated the eggs at low elevation (normoxia, 95% O_2_ availability compared to sea level equivalent) or at extreme high elevation (hypoxia, 72% O_2_ availability compared to sea level equivalent). We monitored embryo physiology (heart rates; an indicator of cardiovascular output and a proxy for metabolism in ectothermic amniotes; Crossley & Burggren 2009) and egg mass throughout the incubation. We then measured important aspects of hatchling phenotype (body mass and body size) and two aspects of fitness‐related performance (sprint swimming speed and apnea duration) of the juveniles (Aubret *et al*. 2015). Finally, we tested whether expected deleterious effects of incubation at extreme high elevation would persist after hatchlings are returned to low elevation, which would indicate that changes in physiology and performance are due to remodeling of related pathways beyond the immediate restrictions of oxygen reduction.

## MATERIALS AND METHODS

### Experimental design

**Figure 1:**
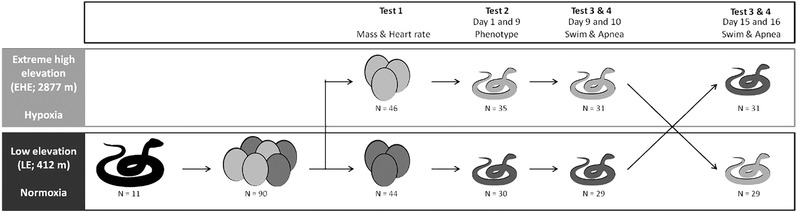
Experimental design. Eggs were collected from gravid females sampled from low elevation viperine snake populations in the foothills of the Pyrenees (432 to 518 m ASL). Within 24 h of oviposition, clutches were evenly split into 2 groups with equal average egg masses. For each clutch, one half‐clutch was transplanted to the extreme high elevation (EHE) laboratory at 2877 m ASL, while the second half‐clutch underwent incubation at low elevation (LE) 436 m ASL. Eggs mass and embryo heart rate were measured throughout incubation (test 1). At hatching, a number of morphometric traits were measured in juveniles (test 2). All hatchlings were tested for swimming and apnea performance in the environment their eggs were incubated (test 3 & 4) and then again after being translocated to the alternative treatment (test 3 & 4).

We captured 12 gravid female viperine snakes along the banks of the Lez River (Department of Ariège, France) between June and July 2016. Capture sites spanned from 432 to 518 m ASL. A total of 113 eggs were obtained between July 8, 2016 and July 28, 2016 (mean clutch size ± SD = 9.4 ± 4.3 eggs). All females were returned to their exact site of capture within 2 weeks of egg‐laying. Twenty‐three eggs were infertile or died within the first 7 days post‐oviposition, leaving 90 eggs from 11 females allocated to 2 treatments for experiments (Fig. 1): low elevation (LE) and extreme high elevation (EHE). The LE treatment was located at the Theoretical and Experimental Ecology Station of Moulis, National Center for Scientific Research (SETE‐CNRS; 42.958394ºN, 1.086440ºE; 436 m ASL; PO_2_ ≈ 20.1 kPa) and the EHE treatment was located at the Observatory Midi‐Pyrénées of the Pic du Midi de Bigorre (42.936389ºN, 0.142472ºE; 2877 m ASL; PO_2_ ≈ 15.3 kPa). This difference in elevation results in a decrease in atmospheric pressure, with associated reduction in the partial pressure of gases, including oxygen, carbon dioxide, and water vapor (Millet & Debevec 2020; Richalet 2020). Most relevant to our hypotheses is the 25% reduction in oxygen availability at the Pic du Midi de Bigorre lab in comparison to sea level (Bouverot 2012; Cordero *et al*. 2017b).

Eggs were weighed using a digital scale to the nearest 0.01 g within 12 h of oviposition, individually marked for identification with a pencil, and allocated to LE and EHE treatments using a split‐clutch design within 24 h of oviposition. Because egg mass influences both embryo metabolism and hatching phenotype (Nelson *et al*. 2004; Aubret 2013), and egg mass varied among clutches (Kruskal–Wallis test: *H* = 61.97, df = 10, *P* < 0.001), eggs were ranked within each clutch from lightest to heaviest and alternately assigned to treatments in order to ensure no difference in egg mass between treatments (Kruskal–Wallis test: *H* = 0.082, df = 1, *P* = 0.774). LE and EHE treatment half clutches were placed in a plastic container (20 cm × 15 cm × 5 cm) on a 2 cm layer of wet vermiculite (1:5 water to vermiculite by volume) and incubated in two identical incubation chambers (ExoTerra Model PT‐2445, Rolf C. Hagen Inc., USA) set at a constant 28 °C, a temperature successfully used for artificially incubating eggs of the viperine snake (Aubret 2013; Aubret *et al*. 2016a, 2017). Water bowls placed within each incubator ensured ambient humidity remained at 100% throughout incubation.

Out of 90 eggs, 65 embryos from 10 females successfully hatched (72.2% hatching success rate) while 25 died at various stages during incubation. Another 5 neonates died shortly after hatching. We measured morphology (see below) and performance (see below) first on all 60 hatchlings at their altitude of incubation (LE or EHE). Nine days post‐hatching (after all yolk was assimilated: Ji *et al*. 1999) hatchlings were tested for swimming performance and at 10 days for apnea performance (see below). At 13 days post‐hatching, LE treatment hatchlings were transferred to EHE while hatchlings from the EHE treatment were brought down to LE. After a 24‐h acclimation period, snakes were tested for swimming and apnea performance at age 15 days and 16 days (Fig. 1). Water temperature was 25 °C for both performance measures because it is within the range of optimal temperature for swimming speed in this species (Hailety & Davies 1986; Aubret *et al*. 2015). Once tests were completed, young snakes were fed and released at the maternal capture site.

### Egg mass and heart rate measurements

We weighed each egg using a digital scale (to the nearest 0.01 g) within 12 h of oviposition, and then every 7 days until hatching (Fig. 1; test 1). Embryo heart rates were first measured at 7 days post‐oviposition and then every 7 days until hatching (Fig. 1; test 1). To measure embryo heart rates, we used the Buddy digital egg monitor (MK2, Avitronics, Cornwall, UK) under the standardized protocol described for eggs. We conducted the measures at the same temperature as incubation (28 °C). Each egg was gently placed on the sensor pad for heart rate reading (a stable reading was obtained after approximately 30 s) and then returned to its clutch. All eggs were only briefly (≤1 min) placed in the digital egg monitor to mitigate potential temperature changes owing to exposure to infrared sensors (Sartori *et al*. 2015; Hulbert *et al*. 2017). While embryonic heart rates are correlated with rates of oxygen consumption in snake and lizard embryos (Souchet J. & Gangloff E. J., unpublished data; Kouyoumdjian *et al*. 2019), we note that change in heart rate is but one of several physiological mechanisms important for the maintenance of energy flux (Sartori *et al*. 2017).

### Hatchling measurements

Hatching occurred between August 20, 2016 and September 8, 2016 and hatchlings were individually marked for identification by the hot branding technique on the ventral scales (Winne *et al*. 2006)) within 24 h of emergence. Hatchlings were weighed using a digital scale (to the nearest 0.01 g), measured for snout–vent length (SVL) using a measuring tape (to the nearest 0.1 mm), and sexed via hemipene eversion (Fig. 1; test 2). While sex is genetically determined in snakes and so we did not expect an effect of treatment on sex determination, we tested for differential effects of treatments between the sexes in developing embryos which could result in skewed hatchling sex ratio. We also weighed the yolk leftover in the eggshell (residual egg yolk) using a digital scale (to the nearest 0.01 g). Juveniles were housed together by hatching date in plastic containers (15 cm × 10 cm × 5 cm) with a water dish, shelter, and paper towel as a substrate in incubation chambers (ExoTerra Model PT‐2445, Rolf C. Hagen Inc.) set at constant 20 °C. While below the optimum temperature for performance, this temperature was chosen because it provides high levels of survival and growth for juveniles of this species (J.S., unpublished personal data). Juveniles were measured again at 9 days post‐hatching for SVL and body mass prior to performance testing. We also calculated body condition as the residual of the log_10_‐mass on log_10_‐SVL linear regression at hatching day and at 9 days post‐hatching.

### Swimming performance

For this test, we were interested in measuring the maximal sprint swimming speed to evaluate the potential limitation of hypoxia on this ecologically‐relevant performance. To estimate sprint swimming performance, we used a procedure that has been validated for snakes (Shine & Shetty 2001; Aubret 2004; Aubret *et al*. 2005a), modified for juvenile viperine snakes. A high‐definition wide‐angle digital camera (25 fps, Sony Model HDR‐XR160E, Sony Corporation) was fitted above a linear 100 cm × 20 cm × 20 cm swimming track and used to record swimming trials (Fig. 1; test 3). The tank was filled to a depth of 5 cm with water maintained at 25 °C using aquarium heaters. Each snake swam 10 consecutive lengths. Raw data were extracted from video files with the software Tracker (Brown 2019). The fastest performance over 10 cm from all trials (sprint swimming speed) was utilized for swimming analysis.

### Apnea performance

To test for maximum voluntary breath‐holding (Fig. 1; test 4), we used the procedure described in Aubret *et al* (2015). Briefly, a glass aquarium (25 cm × 15 cm × 20 cm) was filled with 20 cm of water maintained at 25 °C. Up to 4 snakes were tested simultaneously. Snakes were presented to the open end of a tube (opaque PVC tubes 10 cm in length and 2 cm in diameter, closed at one end and ballasted to ensure stability under water). As soon as the snake voluntarily entered the tube, the unit was fully immersed in the water and tilted upward to make sure no air bubbles remained trapped. The tubes were then oriented toward the side of the aquarium, facing the observer, with the tube opening in direct contact with the glass. This allowed the observer to monitor the movement of the snakes inside the tube. When snakes made contact between their snout and the glass, the observer gently knocked the glass with the tip of a finger to scare them back down into the tube. This stimulus, repeated as long as necessary, encouraged the animal to prolong the duration of its time in the safety of the tube, presumably until its need to breathe overcame the perceived risk of predation imposed by the observer. At this point, the juvenile pushed against the glass with its snout and moved the tube away from the glass, allowing the snake to exit. The time taken from immersion to surface was recorded with digital chronometers (±1 s).

### Data analysis

We first assessed the influence of LE and EHE treatments and time on two measures of embryo development (test 1): egg mass and heart rate. We used linear mixed‐effect models, including as main effects treatment (LE or EHE), age at measurement (in days after hatching, treated as a categorical effect), and their interaction. Then, we assessed the influence of both treatments on 10 measures of hatchling phenotypes (test 2): survival to hatching, sex ratio, incubation time, residual egg yolk, body mass at 1 day and 9 days post‐hatching, body size (SVL) at 1 day and 9 days post‐hatching, and body condition at 1 day and 9 days post‐hatching. For survival to hatching and sex ratio, we used generalized linear mixed models, and for the 8 other tests, we used linear mixed‐effect models, including in all models the main effects of treatment. We also assessed the influence of treatment on the two measures of performance: sprint swimming speed (test 3) and apnea time (test 4). We used linear mixed‐effect models, including the main effects of treatment (LE or EHE), the location of the test (low elevation or extreme high elevation), sex (male or female), and the covariates of body size (SVL) for swimming performance or body mass for apnea performance.

To meet assumptions of normal distribution of residuals, we square root transformed egg mass and apnea time. To account for the non‐independence of siblings, we included the clutch of origin as a random effect in all models. In models for which we measured individuals repeatedly (egg mass, heart rates, sprint swimming speed, and apnea time), we also included individual as a random effect. We used type III sums of squares to assess the significance of main effects, incorporating a Kenward–Roger denominator degree of freedom approximation (Kenward & Roger 1997). All analyses were conducted with the lme4 package (Bates *et al*. 2014) and figures were made with the ggplot2 package (Wickham 2016) in the programming language R 3.4.3 (R Development Core Team 2017).

## RESULTS

### Egg mass variation and embryonic heart rates

**Figure 2:**
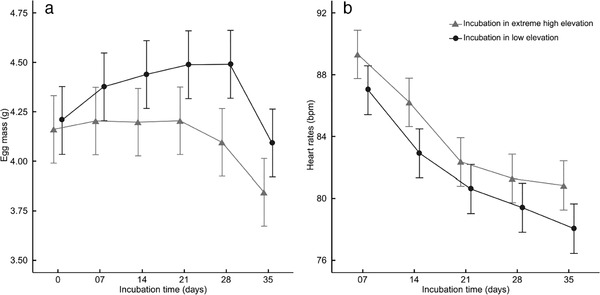
Egg mass (a) and embryo heart rate (b) through incubation time in viperine snakes at low elevation (LE; *N* = 44; circle) and extreme high elevation (EHE; *N* = 46; triangle). Least‐squares means ± SE estimated by linear mixed models are plotted.

Elevation treatments significantly altered egg mass trajectories (Table 1, Fig. 2a). Eggs incubated at LE maintained higher mass across the incubation period compared to eggs incubated at EHE. Further, the drop in egg mass prior to hatching was sharper in the LE treatment (−8.69% mass change between 28 days and 35 days) compared to eggs in the EHE treatment (−6.34% mass change between 28 days and 35 days; Fig. 2a). Eggs incubated at LE gained mass until 28 days before decreasing, while eggs incubated at EHE maintained their initial mass until 21 days before decreasing (Table 1). Nevertheless, the eggs from EHE lost more mass between oviposition and hatching compared with eggs from LE (mass loss of −7.69% and −2.38%, respectively). Heart rates from both incubation treatments followed a similar trend across the incubation period (Fig. 2b). Heart rates decreased throughout incubation but remained consistently and significantly higher in embryos incubated at EHE (Table 1).

**Table 1:** Results of linear mixed‐effect models testing for the effect of incubation treatment (LE or EHE), age at measurement (day post‐hatching), and their interaction on embryo developmental parameters in eggs of the viperine snake

	Egg mass	Heart rates
	LE (*N* = 44); EHE (*N* = 46)	LE (*N* = 44); EHE (*N* = 46)
Day	*F*_5, 437.43_ = 2707; *P* < 0.001^***^	*F*_4, 348.50_ = 24.71; *P* < 0.001^***^
Treatment	*F*_1, 78.37_ = 7.62; *P* = 0.007^**^	*F*_1, 77.92_ = 15.36; *P* < 0.001^***^
Day × Treatment	*F*_5, 437.06_ = 4.59; *P* < 0.001^***^	*F*_4, 348.46_ = 0.26; ***P* = 0.924**

Sample numbers (*N*) for low elevation (LE) and extreme high elevation (EHE) treatments are indicated under the developmental parameters. Significant factors shown in bold with one (*P* < 0.05), two (*P* < 0.01), or three (*P* < 0.001) asterisks.

### Hatching success and morphological measurements

Hatching success (test 2) did not differ significantly between embryos incubated at LE versus EHE (68.2% vs. 76.1% success, respectively; χ² = 2.31, df = 1, *P* = 0.128). Hatchling sex ratio did not differ significantly between embryos incubated at LE versus EHE (50% vs. 62.9% females, respectively; χ² = 1.11, df = 1, *P* = 0.293). Embryos incubated at LE had on average a longer incubation time (by 2%) compared to embryos incubated at EHE (Table 2). LE eggs also produced heavier hatchlings (by 9%; Table 2), although hatchlings did not differ in length or body condition (Table 2). LE embryos assimilated more yolk than embryos incubated at EHE (i.e. had 44% less residual yolk; Table 2). At 9 days post‐hatching, juveniles from embryos incubated at LE were significantly longer (by 3%; Table 2) than juveniles incubated at EHE. On the other hand, body mass and body condition at 9 days did not differ between treatments (Table 2).

**Table 2:** Differences in hatchling traits over the first 9 days of post‐hatching life between juvenile viperine snakes incubated at low elevation (LE) and at extreme high elevation (EHE)

	LE	EHE	*F* (dfn, dfd)	*P*
Incubation time (days) LE (*N* = 30); EHE (*N* = 35)	44.77 ± 1.27	44.03 ± 1.29	20.43 (1, 54.50)	< 0.001^***^
Body mass (g) at 1 day LE (*N* = 30); EHE (*N* = 35)	2.95 ± 0.50	2.71 ± 0.52	10.12 (1, 55.04)	0.002^**^
Body size (cm) at 1 day LE (*N* = 30); EHE (*N* = 35)	14.83 ± 0.73	14.53 ± 1.14	2.03 (1, 56.57)	0.159
Body condition at 1 day LE (*N* = 30); EHE (*N* = 35)	0.01 ± 0.05	−0.01 ± 0.04	3.08 (1, 56.64)	0.084
Residual egg yolk (g) LE (*N* = 30); EHE (*N* = 35)	0.25 ± 0.15	0.45 ± 0.49	4.64 (1, 58.88)	0.035^*^
Body size (cm) at 9 days LE (*N* = 30); EHE (*N* = 34)	15.52 ± 0.79	15.09 ± 0.91	8.77 (1, 54.13)	0.005^*^
Body mass (g) at 9 days LE (*N* = 30); EHE (*N* = 34)	2.07 ± 0.41	1.98 ± 0.35	2.16 (1, 54.45)	0.147
Body condition at 9 days LE (*N* = 30); EHE (*N* = 34)	0.008 ± 0.049	−0.005 ± 0.042	0.52 (1, 54.23)	0.472

Linear mixed‐effect models were used to test the effects of treatment on the relevant traits. Raw means ± SD are given. Significant factors shown in bold with one (*P* < 0.05), two (*P* < 0.01), or three (*P* < 0.001) asterisks.

### Effects of incubation treatment and translocation on swimming performance of juveniles

**Figure 3:**
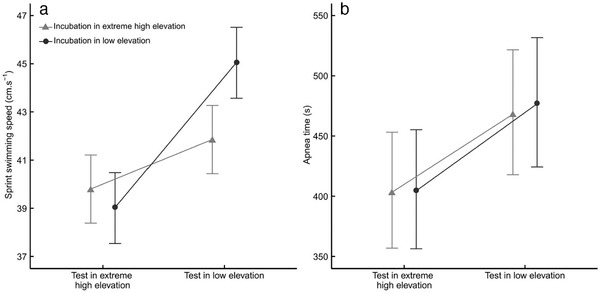
Sprint swimming speed (a) and apnea performance (b) by incubation treatment (LE; *N* = 29; circle and EHE; *N* = 31; triangle) and test location (low elevation and extreme high elevation) in viperine snakes. Least‐squares means ± SE estimated by linear mixed models are plotted.

All snakes showed higher sprint swimming speed (test 3) at LE rather than EHE (Table 3, Fig. 3a). The swimming speed of snakes incubated at EHE increased after being translocated to LE (5% faster), while the swimming speed of snakes incubated at LE decreased after translocation to EHE (13% slower). This is demonstrated by the significant interaction of incubation treatment and test location (Table 3): Snakes incubated at LE exhibited higher performance at LE, but groups did not differ at EHE. As expected based on other studies of snake swimming speed (Shine & Shetty 2001), longer snakes swam faster than smaller snakes (Table 3).

**Table 3:** Results of linear mixed‐effect models testing the determinants of performance in juvenile viperine snakes. Sample numbers (N) for both low elevation (LE) and extreme high elevation (EHE) treatments are indicated under the performance tested

	Sprint swimming speed	Apnea time
	LE (*N* = 29); EHE (*N* = 31)	LE (*N* = 29); EHE (*N* = 31)
Test location	*F*_1, 58.00_ = 16.82; *P* < 0.001^***^	*F*_1, 58.00_ = 4.49; *P* = 0.038^*^
Treatment	*F*_1, 51.94_ = 1.01; *P* = 0.319	*F*_1, 49.92_ = 0.01; *P* = 0.920
Test location × Treatment	*F*_1, 58.00_ = 4.06; *P* = 0.048^*^	*F*_1, 58.00_ = 0.01; *P* = 0.912
Sex	*F*_1, 52.22_ = 0.74; *P* = 0.392	*F*_1, 51.60_ = 0.64; *P* = 0.428
Body size (cm) at 9 days	*F*_1, 42.83_ = 15.86; *P* < 0.001^***^	—
Body mass (g) at 9 days	—	*F*_1, 53.86_ = 4.72; *P* = 0.034^*^

Significant factors shown in bold with one (*P* < 0.05), two (*P* < 0.01), or three (*P* < 0.001) asterisks.

### Effects of incubation treatment and translocation on apnea performance of juveniles

There was no effect of treatment on apnea performance (test 4), while snakes for both groups showed higher apnea performance at LE rather than EHE (15% longer; Table 3, Fig. 3b). Additionally, body mass influenced apnea performance, with lighter snakes holding their breath for longer durations (Table 3).

## DISCUSSION

Our study is intended to quantify the restrictions imposed by transplantation to extreme high elevation and the potential limits of organismal responses to these constraints, relevant in the current context of global warming. We explored the way egg incubation and hatching success (primary components of successful population establishment during colonization processes) were affected by extreme high elevation (i.e., hypoxia) compared to control eggs (incubated at low elevation) in the viperine snake. Although the EHE treatment did not significantly alter hatching success, it generated significant differences in egg development and affected hatchling phenotypes, including performance decrements that persisted after translocation back to the native elevation.

### Embryo development and hatchling measurements

Typical physiological adjustments to hypoxia in other taxa include suppressed embryo metabolism, often measured as reduced heart rate (Laughlin 1978; Monge & Leon‐Velarde 1991; Crossley & Altimiras 2005; Crossley & Burggren 2009; Du *et al*. 2011; Cordero *et al*. 2017a, 2011; Kouyoumdjian *et al*. 2019). However, heart rates of developing viperine snake embryos exhibited the opposite trend: their heart rates increased at EHE (Fig. 2b). This is a puzzling result and a physiological response that is opposite to what is observed in other taxa (see above references). Further, while eggs incubated at LE tended to gain mass during incubation, eggs incubated at EHE maintained their mass over the same period (Fig. 2a), suggesting a low efficiency of water or carbon dioxide diffusion (Cunningham & Hurwitz 1936). Excessive water loss in snake eggs may gradually increase yolk viscosity and impede absorption by the developing embryo (Cunningham & Hurwitz 1936; Aubret *et al*. 2005b). Eggs exposed to EHE, by losing excessive water, may have exposed the embryo to a similar constraint, leading to lesser yolk intake (and higher amounts of residual yolk post‐hatching) and consequently smaller body size at hatching (Table 2). These results collectively suggest that either higher metabolic rates (i.e. heart rates), excessive water loss (rendering the yolk hard to assimilate), or a combination of both, generated early hatching at EHE (Spencer *et al*. 2001; Du *et al*. 2009) compared to sibling eggs incubated at LE. Further investigations will also be needed to ascertain whether higher heart rates in EHE embryos resulted from exposure to hypoxia (a counter‐intuitive finding, see references above) or from excessive water loss causing physiological stress to the embryos.

Because the difference in incubation time was minimal between the two treatment groups (i.e. <24 h; Table 2), one could question the biological relevance of this effect on hatching fitness and long‐term survival prospects. While further investigations are needed to address this question, there is evidence that incubation times (at 28 °C) are heavily constrained in the viperine snake (i.e. always remain within a 24 h boundary, irrespective of experimental treatments; Aubret *et al*. 2016a,b, 2017) and early hatching may entail deleterious effects. For example, early hatched Japanese quail chicks (*Coturnix coturnix japonica*) take 1–2 h longer to stand than normal chicks (Vince & Chinn 1971), while early hatched turtles (*Chrysemys picta*) showed reduced neuromuscular function for at least 9 months after hatching (Colbert *et al*. 2010). Nevertheless, in areas where growing seasons are short (such as at high elevation), hatching early can be advantageous to respond to temporal constraints on food acquisition (Edge *et al*. 2017). In our study, however, early hatching is combined with a lesser ability to absorb egg yolk, smaller body size at hatching, poorer body condition at hatching, and slower growth rates (Table 2). These results are consistent with metabolic compensation, a physiological mechanism whereby stressful incubation conditions generate faster paces of development (McGlashan *et al*. 2012; McGlashan *et al*. 2015; Aubret *et al*. 2016b). Further, or alternatively, low partial pressure of O_2_ at high altitude (>2000 m ASL) is known to render embryonic development challenging due to aerobic energetic restrictions in converting egg energy (yolk) into tissue (Wangensteen *et al*. 1974; Rahn *et al*. 1977; Bouverot 1985; Monge & Leon‐Velarde 1991; Noble 1991; Vleck & Hoyt 1991; Vleck & Vleck 1996; León‐Velarde & Monge 2004). Importantly, change in heart rate is one of many possible compensatory physiological mechanisms to accommodate abiotic limitations and may, in itself, not represent increased metabolism (Sartori *et al*. 2017). Whether or not metabolic compensation or a comparable physiological mechanism operated in embryos incubated at EHE remains unclear at this stage and will warrant future investigation. Importantly though, EHE did not prevent eggs from developing and hatching altogether, as hatching success did not differ between the two treatments groups (LE: 68.2% vs. EHE: 76.1%; see Results). Nevertheless, EHE altered body size in neonate snakes as well as post‐hatching growth rates, both important fitness proxies in squamates (Kissner & Weatherhead 2005; Mayer *et al*. 2016; Gangloff *et al*. 2018). However, the long‐term adaptive potential for observed changes in development and physiology has yet to be tested.

### Swimming and apnea performance

Our results show that EHE significantly affected hatchling swimming performance, but not apnea performance. This difference persisted even after translocation to low elevation, suggesting a genuine long‐term change of physiological and performance capacity. EHE juveniles, when transferred back to LE, did not recover full performance compared to their siblings from the LE treatment. Further, juveniles incubated at EHE did not perform better than the LE siblings when tested at EHE (Fig. 3a). These findings suggest that (i) snakes’ physiology was impaired during development (muscle function, locomotion, or cardiorespiratory capacity) beyond a simple reduction of body size at birth and that (ii) physiology and body size were affected in a way that did not enhance organismal function in hypoxic conditions. As a result, such morphological and physiological shifts are likely a mechanistic consequence of development in hypoxic conditions, considered developmental constraints rather than an acclimation effect and thus non adaptive (Bennett 1997; Forsman 2015).

### General conclusion

It should be kept in mind that our experiment did not aim at mimicking a biologically relevant situation: These organisms are unlikely to climb over 2500 m (i.e. the distance separating origin populations from the extreme high elevation treatment) along the altitudinal gradient to breed. Any range shift driven by climate change is likely to be gradual, potentially allowing for animals to adjust their physiology and behavior by means of phenotypic plasticity and natural selection acting on advantageous genetic variants (mixed selection on plastic and non‐plastic attributes over different time scales, eventually leading to local adaptation; Rezende *et al*. 2005; Beall 2006; Hammond *et al*. 2006; Powell & Hopkins 2010; Storz *et al*. 2010; Mueller *et al*. 2015). Indeed, several squamate species have adapted to permanent life at extreme high elevations (i.e. Atlas Day gecko, *Quedenfeldtia trachyblepharus*: Bouazza *et al*. 2016; *Liolaemus* lizards: Marquet *et al*. 1989; Qinghai toad‐headed lizards, *Phrynocephalus vlangalii*: Wu *et al*. 2018; western fence lizard, *Sceloporus occidentalis* and sagebrush lizard, *Sceloporus graciosus*: Adolph 1990; Pyrnean rock lizard, *Iberolacerta bonnali*: Pottier 2012). These records are testimony that colonization and life at extreme high altitude is possible for oviparous ectotherm amniotes, although the interactions between elevation, colonization dynamics, warming speed, plasticity, and local adaptation remain to be understood. Our study shows that extreme high elevation colonization by the viperine snake will not be prevented, but likely slowed down by hypoxia. Notably, in the context of global warming, it will be essential to measure how a combination of different environmental factors might interact to affect development and performance. For example, the effects of oxygen level may manifest differently depending on temperatures, with the effects of reduced oxygen availability stronger at high temperatures (Gangloff & Telemeco 2018). These impacts could in turn limit the ability to colonize higher elevation. As a start, our study demonstrates that extreme high elevation has significant effects on embryo development and hatchling phenotypes, prompting further research on the matter, specifically on the interactive effects of oxygen levels and temperature.

## AUTHOR CONTRIBUTIONS

JS and FA contributed to experimental design and logistics. JS, GM, CB, and FA conducted experiments. JS, EJG, and FA conducted statistical analyses. JS, EJG, FA, AT, RB, JC, OC, OG, AMS, ED, HLC, MMT, LB, GP, and HP drafted the manuscript. All authors contributed critically to the drafts and gave final approval for publication.
